# Longevity and population age structure of the arroyo southwestern toad (*Anaxyrus californicus*) with drought implications

**DOI:** 10.1002/ece3.4158

**Published:** 2018-05-20

**Authors:** Robert N. Fisher, Cheryl S. Brehme, Stacie A. Hathaway, Tim E. Hovey, Manna L. Warburton, Drew C. Stokes

**Affiliations:** ^1^ Western Ecological Research Center US Geological Survey San Diego California; ^2^ California Department of Fish and Wildlife Santa Clarita California; ^3^ Present address: San Diego Natural History Museum San Diego California

**Keywords:** amphibian, amphibian decline, endangered species, life span, skeletochronology, southern California

## Abstract

The arroyo southwestern toad is a specialized and federally endangered amphibian endemic to the coastal plains and mountains of central and southern California and northwestern Baja California. It is largely unknown how long these toads live in natural systems, how their population demographics vary across occupied drainages, and how hydrology affects age structure. We used skeletochronology to estimate the ages of adult arroyo toads in seven occupied drainages with varying surface water hydrology in southern California. We processed 179 adult toads with age estimates between 1 and 6 years. Comparisons between skeletochronological ages and known ages of PIT tagged toads showed that skeletochronology likely underestimated toad age by up to 2 years, indicating they may live to 7 or 8 years, but nonetheless major patterns were evident. Arroyo toads showed sexual size dimorphism with adult females reaching a maximum size of 12 mm greater than males. Population age structure varied among the sites. Age structure at sites with seasonally predictable surface water was biased toward younger individuals, which indicated stable recruitment for these populations. Age structures at the ephemeral sites were biased toward older individuals with cohorts roughly corresponding to higher rainfall years. These populations are driven by surface water availability, a stochastic process, and thus more unstable. Based on our estimates of toad ages, climate predictions of extreme and prolonged drought events could mean that the number of consecutive dry years could surpass the maximum life span of toads making them vulnerable to extirpation, especially in ephemeral freshwater systems. Understanding the relationship between population demographics and hydrology is essential for predicting species resilience to projected changes in weather and rainfall patterns. The arroyo toad serves as a model for understanding potential responses to climatic and hydrologic changes in Mediterranean stream systems. We recommend development of adaptive management strategies to address these threats.

## 1 INTRODUCTION

**Figure 1:**
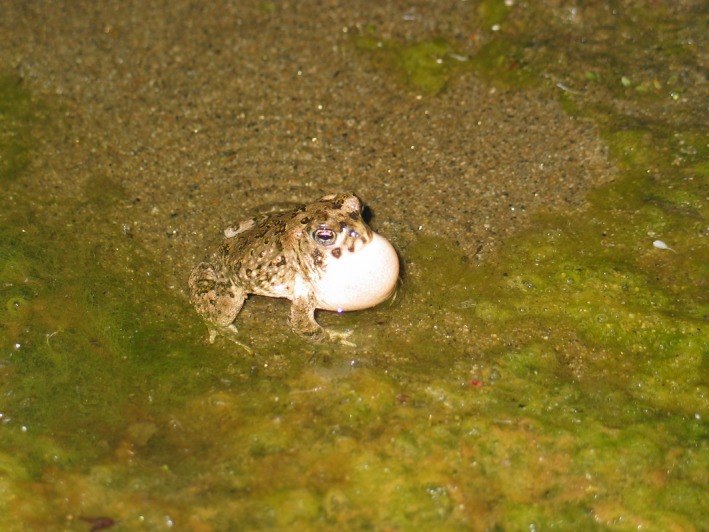
The arroyo southwestern toad (*Anaxyrus californicus*)

Amphibians are declining rapidly on national and global scales; however, some species are declining more rapidly than others (Adams et al., 2013; Beebee & Griffiths, 2005; Lannoo, 2005; Stuart et al., 2004). The arroyo southwestern toad (*Anaxyrus californicus*; arroyo toad; Figure 1) has been extirpated from approximately 75% of its historical habitat and is one of the most vulnerable amphibian species in California (Jennings & Hayes, 1994; Sweet & Sullivan, 2005). The US Fish and Wildlife Service (USFWS) listed the arroyo toad as an endangered species in December 1994 (USFWS, 1994) and released a Recovery Plan in 1999 (USFWS, 1999). The arroyo toad decline has been attributed to extensive habitat loss, human modification to water flow regimes, and the introduction of non‐native predators (US Fish and Wildlife Service, 1999). Climate variability and extremes can affect all of these factors.

The arroyo southwestern toad is a specialized amphibian endemic to the coastal plains and mountains of central and southern California and northwestern Baja California (Ervin, Beaman, & Fisher, 2013; Jennings & Hayes, 1994). It primarily inhabits low gradient streams and rivers containing sandy soils with sandy streamside terraces (Barto, 1999; Sweet, 1992, 1993; Treglia, Fisher, & Fitzgerald, 2015). Reproduction is dependent on the availability of shallow, still, or low flow pools in which breeding, egg laying, and larval development occur. Annual rainfall in southern California is highly variable and heavily influenced by the El Niño–Southern Oscillation (ENSO) cycle (Schonher & Nicholson, 1989). The surface hydrology of stream systems occupied by arroyo toads varies from being ephemeral, where surface water is present only in normal to high rainfall years, to seasonally predictable, where surface water is typically present during the breeding season every year.

California recently experienced an unprecedented drought from 2012 through 2015 (Diffenbaugh, Swain, & Touma, 2015; Funk, Hoell, & Stone, 2014; Griffin & Anchukaitis, 2014; Robeson, 2015). These types of extreme and prolonged drought events have been widely predicted to increase across California and the southwestern United States according to recent climate modeling and climate prediction studies (Seager et al., 2007; Cayan et al., 2010; Dettinger & Cayan, 2014; Diffenbaugh et al., 2015). Hydrological models have linked these climate model projections to significantly reduced streamflow and increased frequency of drying events, particularly in ephemeral stream systems (Jaeger, Olden, & Pelland, 2014; Seager et al., 2013), resulting in potential deleterious effects on the persistence of native fish and invertebrates (Jaeger et al., 2014; Montgomery et al., 2015).

Recent monitoring studies have shown that seasonal hydrology is extremely important in determining arroyo toad population dynamics and the relative risk of stressors such as non‐native species and climate extremes (Brehme, Matsuda, & Fisher, 2013; Miller, Brehme, Hines, Nichols, & Fisher, 2012). Although there is growing knowledge about habitat relationships and stressors (Mitrovich, Gallegos, Lyren, Lovich, & Fisher, 2011), there is still a lack of basic life history information important for modeling population viability and assessing species status. These information gaps include how long toads live in natural systems and how their population structure varies across occupied drainages. Stable age structures and high longevity would indicate arroyo toad populations are more resilient to temporal fluctuations within suitable breeding habitat.

In this study, our primary goals were to determine how long arroyo toads live in natural systems and whether population age structures varied across occupied river and stream systems so that impacts of long‐term climate patterns can be better assessed. For this, we used skeletochronology to estimate the ages of adult arroyo toads in seven occupied drainages with varying surface water hydrology in southern California. This technique is cost efficient in comparison with long‐term capture–recapture studies because it allows for estimation of population age structures in a single year and with single captures of individuals.

## 2 MATERIALS AND METHODS

### 2.1 Study sites

**Figure 2:**
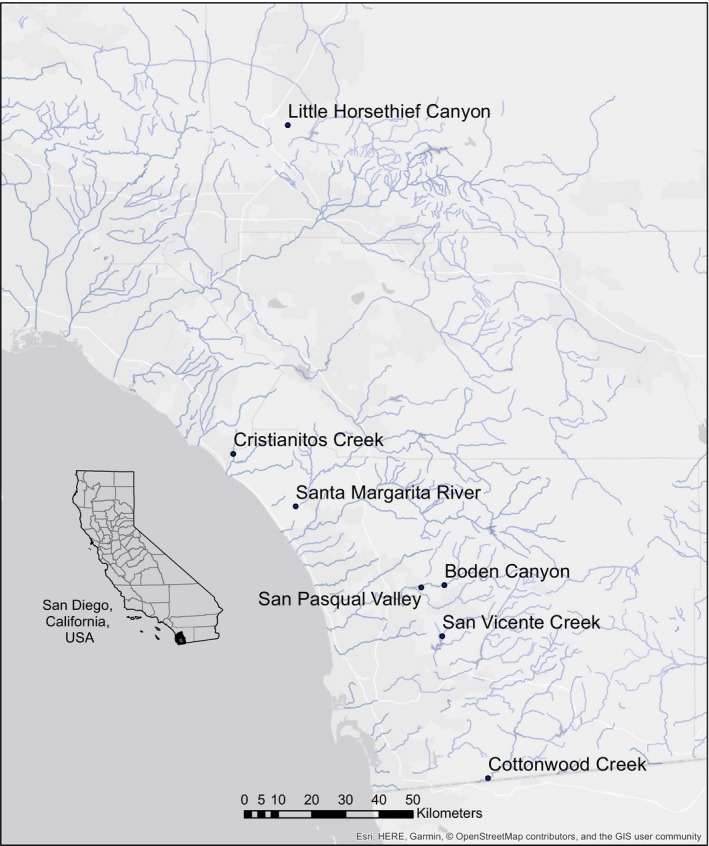
Arroyo toad study sites in southern California

Southern California has a Mediterranean climate with relatively warm, dry summers and mild winters. The rainy season typically falls between October and April with most precipitation occurring in January, February, and March. Rainfall is highly variable among years but averages 263 mm in San Diego and 407 mm in San Bernardino. Our seven survey sites included two with perennial or seasonally predictable water availability (Santa Margarita River, Little Horsethief Canyon) and five ephemeral systems where the presence of water is dependent upon adequate rainfall (Boden Canyon Ecological Reserve, Cottonwood Creek, Cristianitos Creek, San Pasqual Valley, and San Vicente Creek; Figure 2).

### 2.2 Sampling

During the spring and summer of 2003 and 2004, we conducted nighttime arroyo toad surveys at all population sites in accordance with the USFWS protocol (US Fish and Wildlife Service, 1999) with the exception of San Pasqual Valley, Cottonwood Creek, and San Vicente Creek that were sampled in 2003 only. Nocturnal surveys entailed walking along creek and river reaches, covering both aquatic and adjacent terrestrial habitats, in search of adult arroyo toads using visual observation and aural detection of calling males. All surveys were conducted by USGS biologists familiar with the arroyo toad. High‐intensity spotlights were used to provide the required amount of illumination to maximize detection. When we detected adult arroyo toads, we measured snout‐to‐urostyle length (SUL; with the exception of toads in amplexus). For all individuals greater than or equal to 45 mm, we recorded sex and weight and clipped the fourth toe on the right hind limb at the joint just below the third phalange. If the toad was not previously marked with a passive integrated transponder (PIT) tag, we inserted a PIT tag following standard procedures for amphibians (Donnelly, Guyer, Juterbock, & Alford, 1994). When toads that had been previously marked in 2003 were located during 2004, a second toe was removed via toe clipping of the fourth toe on the left hind foot. Clipped toes were preserved in 95% ethanol. Only the first capture of each individual toad was used in the analyses; however, recaptures of toads PIT tagged for this study or by previous researchers allowed us to assess results of skeletochronology age estimates from consecutive years.

### 2.3 Skeletochronology

Skeletochronology involves the aging of individuals by analyzing cross sections of long bones, such as phalanges, for the presence of concentric rings, called annuli or lines of arrested growth (LAG). LAGs are formed in response to seasonal periods of decreased bone growth such as during winter or summer periods of inactivity depending on the species (Bastien & Leclair, 1992; Monnet & Cherry, 2002; Trenham, Shaffer, Koenig, & Stromberg, 2000). Gary Matson's laboratory in Montana, USA, analyzed the arroyo toad toe clips using standardized techniques (McCreary, Pearl, & Adams, 2008). This involved preparing cross sections of the toes, examining the cross sections under a microscope, and counting lines of arrested growth (LAG) in cross sections of the toe bones to estimate age in years. For animals that have a single dormancy period each year, the number of LAGs counted in the cross section of a toe bone should roughly correspond to the number of years the toad has lived. However, it has been shown that counts of LAGs to estimate years of age may be underestimated by 1 to 2 years due to bone resorption of the 1st and 2nd LAGs in some species (Cvetkovici, Tomasevic, Aleksic, & Crnobrnja‐Isailovic, 2005; Fretey & Le Garff, 1996; Plytycz & Bigaj, 1993). Therefore, when number of LAGs was questionable between two consecutive values, the higher value was used in the analyses.

### 2.4 Analysis

We ran regression models in the R statistical computing environment (R Development Core Team, 2008) to investigate the relationship between body size (SUL) and estimated age by skeletochronology. We compared the fit of linear, log‐transformed, and saturation growth rate models with and without sex (male, female) using Akaike's information criterion (AIC) and model selection procedures described by Burnham and Anderson (2002) and used the best fitting model for our analysis. Age structure histograms were also produced in the R environment using ggplot2 (Wickham, 2009).

## 3 RESULTS

A total of 179 individual toads with snout‐to‐urostyle length (SUL) from 45 to 70 mm were detected and processed at the seven sites. We processed 30 toads at Boden Canyon, 41 at Cristianitos Creek, four at Cottonwood Creek, 38 in Little Horsethief Canyon, 18 at San Pasqual Valley, 46 at Santa Margarita River, and two at San Vicente Creek. There were too few animals captured at Cottonwood Creek and San Vicente Creek to assess population age structure, so animals at these sites were used in regression analysis only.

Skeletochronology analysis of toe cross sections from arroyo toads (greater than 45 mm in SUL) resulted in age estimates that ranged from 1 to 6 years across all sites. Five animals PIT tagged in 2003 were also detected and processed in 2004; therefore, we know there is 1‐year age difference between capture events. Skeletochronology estimated correctly that three animals had aged 1 year and estimated the other two animals had aged 0 years (Table 1). In addition, six animals that were previously PIT tagged in 1998 and 2000 as part of previous studies (Holland, Sisk, & Goodman, 2001) were recaptured in 2003 and 2004. We estimated the age upon recapture assuming a minimum age at first capture of 1 year. Based on this assumption, skeletochronology analysis estimated the correct minimum age for two individuals and estimated 1 year below the known minimum age for four individuals (Table 1).

**Table 1:** Skeletochronology age (years) estimates of recaptured toads

	Location	PIT tag no.	Year of 1st Capture	Minimum age (in years) at 1st capture	Age (in years) estimate at 1st capture based on skeletochronology	Year of recapture	Age (in years) estimate at recapture based on age at first capture	Age (in years) estimate at recapture based on skeletochronology	Difference (estimate‐known)
Animals first pit‐tagged as part of skeleto study	Boden Canyon	62621317	2004	1	3	2004	3	3	0
	Boden Canyon	62619882	2003	1	2–3	2004	4	3	−1
	Boden Canyon	62619283	2003	1	2	2004	3	3	0
	Cristianitos creek	44094374	2003	1	4	2004	5	4	−1
	Cristianitos creek	44369298	2003	1	3	2004	4	4	0
Animals pit‐tagged in previous studies	Cristianitos creek	406649725b	1998	1	NA	2004	7	6	−1
	Cristianitos creek	414C581237	1998	1	NA	2004	7	6	−1
	Cristianitos creek	42554E3D59	2000	1	NA	2004	5	4	−1
	Cristianitos creek	425639594F	2000	1	NA	2003	4	4	0
	Cristianitos creek	501C771A7C	2000	1	NA	2004	4	3	−1
	Cristianitos creek	501D302704	2000	1	NA	2003	3	3	0

**Figure 3:**
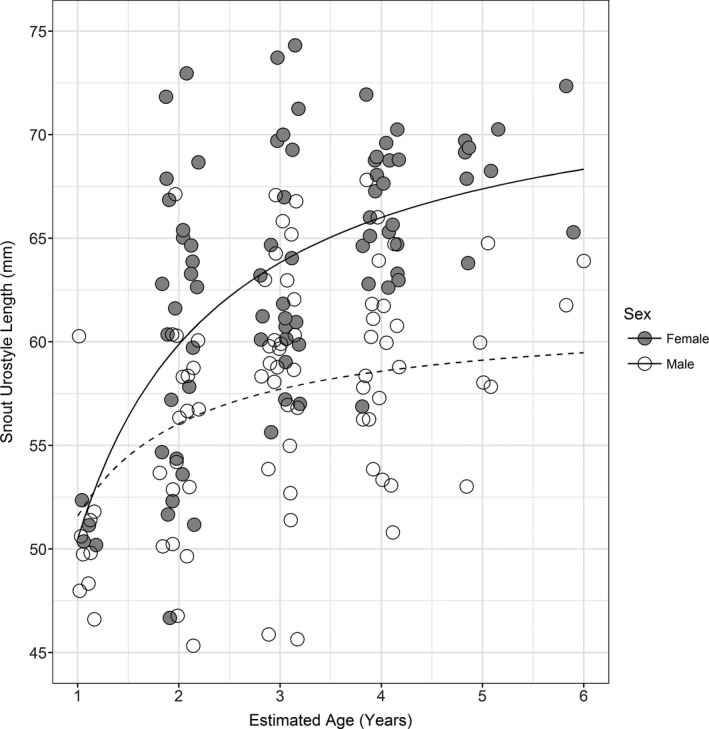
Relationship between size (snout‐to‐urostyle length, mm) and arroyo toad age (years) estimated by skeletochronology

There was a significant association between estimated age by skeletochronology and animal size (snout‐to‐urostyle length; SUL) in both sexes; however, the coefficient of determination showed a much better fit for females (*R* ^2^ = .488, *p* < .001) than for males (*R* ^2^ = .190, *p* < .001). Saturation growth rate equations best represented these data with SUL Length (mm) = (*a* × Age(Years))/(*b* + Age(Years)) where *a* = maximum SUL and *b* = growth rate at 0.5 × *a*. Between the ages of 1 and 6 years, females had an estimated growth rate of 0.46 and average maximum SUL length of 73.5 mm, whereas males had a growth rate 0.19 and an estimated average maximum SUL length of 61.3 mm (Figure 3).

**Figure 4:**
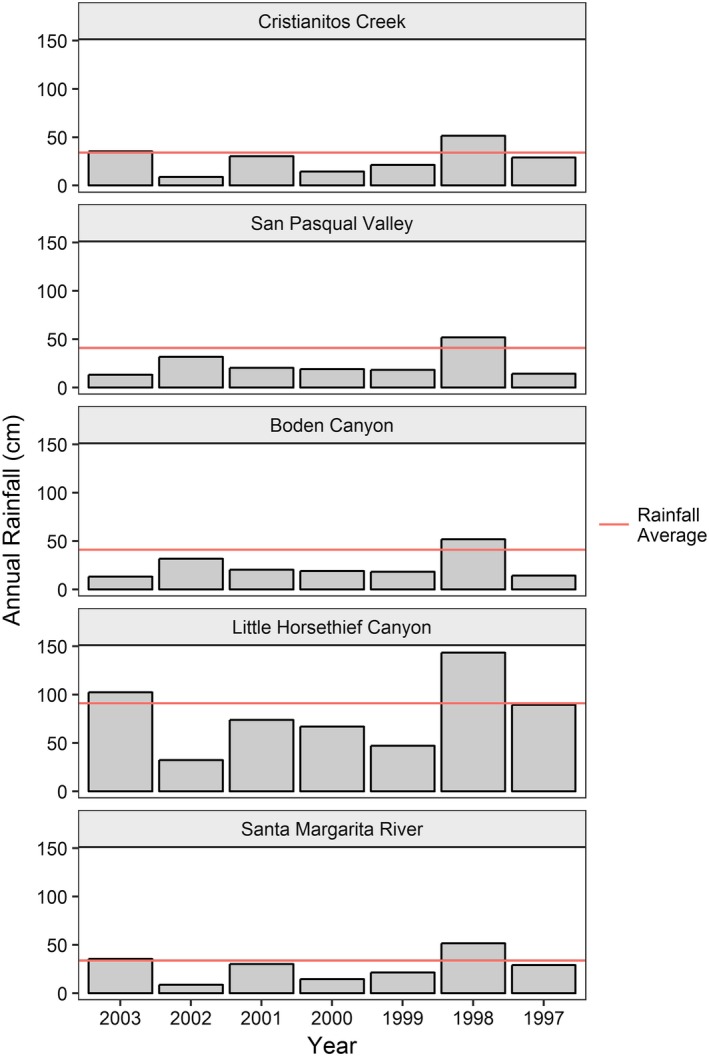
Rainfall (in cm) among years at arroyo toad study sites. “Normal” average annual rainfall indicated by (—) (National CLimate Data Center 2017). (Normal for Little Horsethief Canyon is approximately 91 cm/year, for Santa Margarita River and Cristianitos Creek is ≈34 cm/year, and for Boden Canyon and San Pasqual Valley is ≈41 cm/year (http://cdec.water.ca.gov; https://usclimatedata.com)

**Figure 5:**
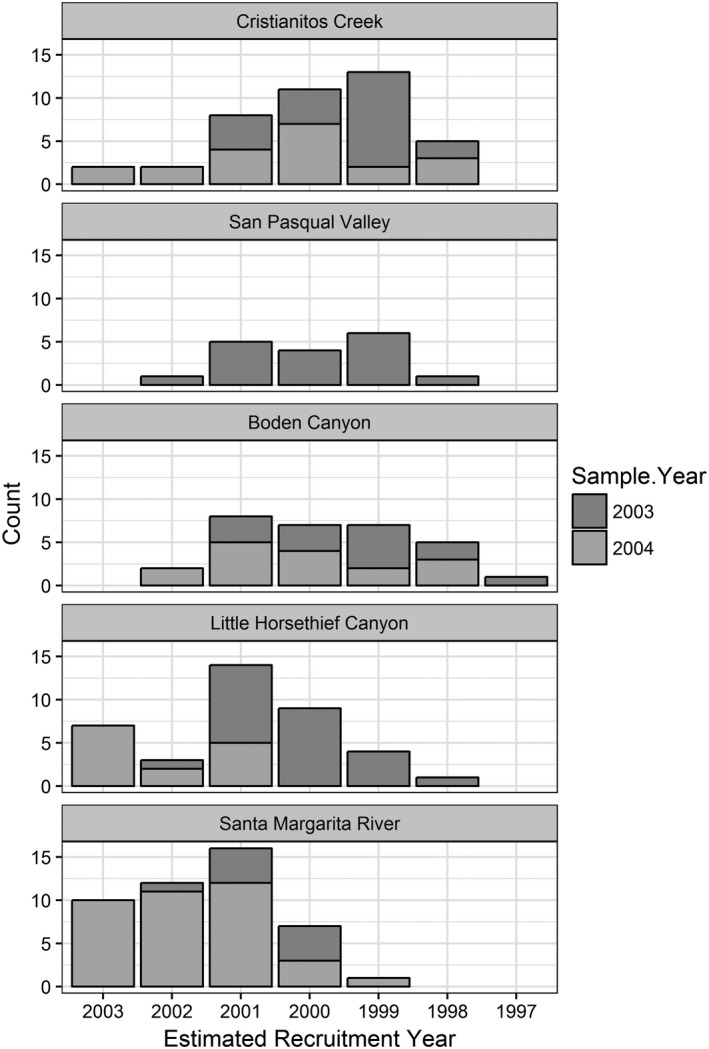
Estimated age distribution of arroyo toads among study sites. (Seasonally predictable sites include Santa Margarita River and Little Horsethief Canyon)

**Figure 6:**
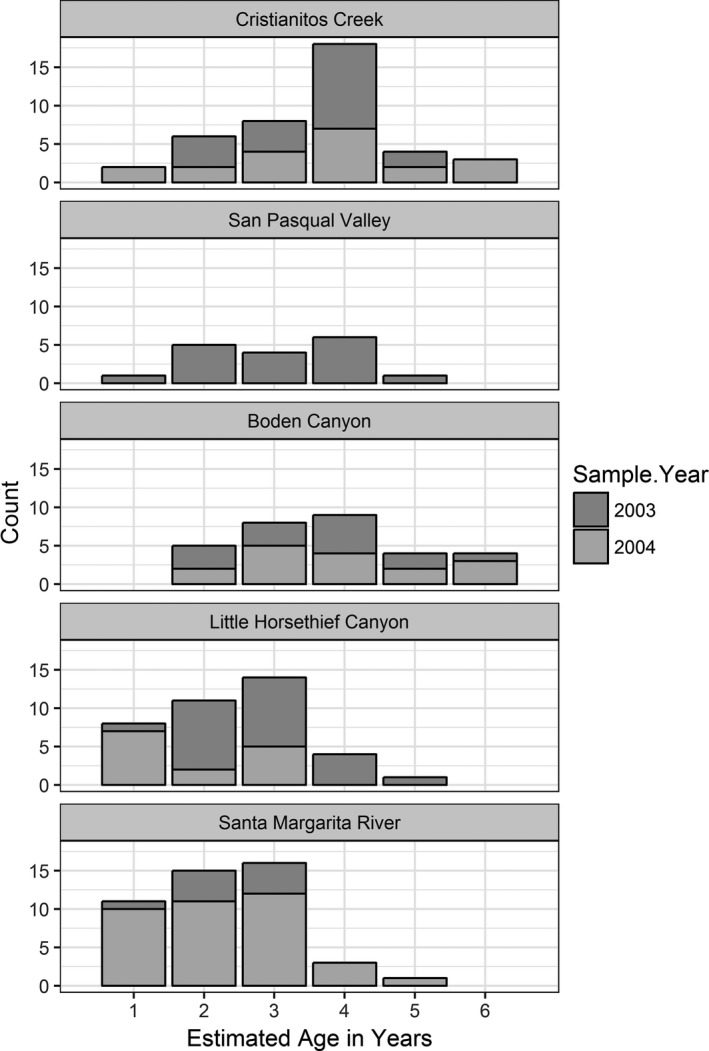
Population age structures across study sites. (Seasonally predictable sites include Santa Margarita River and Little Horsethief Canyon)

Rainfall varied among sites and years. Rainfall records from weather stations within close proximity to our sites (cdec.water.ca.gov; usclimatedata.com; wunderground.com) indicated below normal rainfall for most years at all sites (Figure 4). Population age structures of arroyo toads varied among sites. Our histograms of toad ages show that ephemeral sites (Cristianitos Creek, San Pasqual Valley, and Boden Canyon) were skewed toward individuals ranging 3 to 5 years old, recruited into the populations between 1998 and 2001 (Figures 5 and 6). In contrast, ages of toads at sites with seasonally predictable surface water during the breeding season (Little Horsethief Canyon, Santa Margarita River) were skewed toward younger individuals which indicates more consistent recruitment for these populations (Figures 5 and 6).

## 4 DISCUSSION

### 4.1 Age estimation

Skeletochronological analysis was successful in documenting differing age structures among populations. However, the control samples indicated that this method may often underestimate the age of toads by at least 1 year. There are several possible reasons for underestimation. First, juveniles may not form their first LAG in the dry season or winter following metamorphosis (Driscoll, 1999). Second, similar studies on other toad species have shown that one, or less commonly, two early LAGs may become partially to fully resorbed (Cvetkovici et al., 2005; Fretey & Le Garff, 1996; Plytycz & Bigaj, 1993). Also, growth rate decreases as individuals get older, resulting in smaller amounts of bone laid down annually and making recognition of these LAGs more difficult (Leclair & Castanet, 1987). For these reasons, our LAG results should be interpreted as estimation of minimum ages. Actual ages could be zero to two or more years greater, with the greatest bias expected to be in the oldest adults. It is unlikely that this strongly biased our assessment of the relative age structures across populations because any underestimation would be expected to act across populations. It will be important to combine skeletochronology with long‐term capture–recapture studies in order to determine age of first LAG, and deposition and reabsorption rates that may result in bias of LAG age estimates. Western toads have life spans from roughly 8 to 11 years, and Couch's Spadefoot toads live 11 to 13 years (Bull, 2006; Tinsley & Tocque, 1995). Our skeletochronology results show that arroyo toads can live to at least 6 years in natural systems, but for the reasons stated above, their maximum life span is likely closer to 8 years.

Most toads appeared to reach close to their maximum size by the second year, after which the growth rate slowed considerably. We found evidence of sexual size dimorphism with maximum growth of adult females estimated to be 12 mm greater than adult males. Sexual size dimorphism where the female is larger is common in most anurans (Monnet & Cherry, 2002; Shine, 1979; Ziang & Lu, 2013).

### 4.2 Population age structure

In situations of stable recruitment and survivorship, we expect to see population age structures biased toward younger individuals and declining as age increases (Kellner & Green, 1995). This was evident in the age structure of the population within the lower Santa Margarita River where there is seasonally predictable surface water during the breeding season. The population in Little Horsethief Canyon also had a somewhat stable population structure as there are seasonally predictable pools of surface water in all but extreme drought years. Population age structures of arroyo toads from the ephemeral streams, however, were skewed toward older adults suggesting that populations in these systems are likely unstable and dependent upon successful reproduction and survival of cohorts from higher rainfall years (Tinsley & Tocque, 1995). This was expected as there is no surface water available for breeding and recruitment in years of below normal rainfall. Older age cohorts roughly corresponded to higher rainfall years in the ephemeral creeks in our study. However, imprecision of age estimates due to unknown variations in LAG deposition and resorption prevents precise identification of specific years of recruitment (Hemelaar, 1985; Leclair & Castanet, 1987; Wagner et al., 2011).

### 4.3 Hydrologic stochasticity, biological responses, and drought projections

Arroyo toads in the ephemeral watersheds appear to be primarily influenced by stochastic processes (i.e., amount of rainfall), while those in perennial systems appear to be primarily influenced by deterministic processes (i.e., predation, competition, and habitat alteration; e.g., Miller et al., 2012). We expect less temporal variability and increased population persistence within the seasonally predictable systems. However, the threat of extirpation of amphibians by non‐native species predation and associated habitat loss in these systems is an immediate and well‐documented threat (reviewed in Kats & Ferrer, 2003; Miller et al., 2012; Brehme et al., 2013). Systems driven by stochastic processes are expected to be more highly variable among years (Death & Winterbourn, 1994; Ross, Matthew, & Echelle, 1985; Therriault & Kolasa, 2000). The Mediterranean climate and influence from the ENSO cycle in southern California result in highly variable annual rainfall. Consequently, ephemeral creeks may remain dry in low rainfall years and experience extensive flooding and scouring in high rainfall years. The populations in ephemeral habitats are at increased risk of extirpation from a prolonged drought. In the second half of the 21st century, the duration of extreme dry events is projected to increase markedly, with most dry spells lasting longer than 5 years and some lasting up to 12 years (Cayan et al., 2010). Results of our study indicate that toads can live up to six or possibly 7 or 8 years; therefore, an extended drought of six or more years would be expected to result in substantial reductions or extirpation of entire populations due to lack of breeding opportunities, reduced food resources, and prolonged drought stress in adults. Water management for increased human water needs may also increase this risk (Marshall, Robles, Majka, & Haney, 2010). Historically, recolonization of suitable habitat from nearby populations of arroyo toads could eventually occur after these unlikely events; however, many current arroyo toad populations are effectively isolated due to habitat fragmentation and extensive development in southern California. Therefore, the cumulative effects of changing rainfall patterns on the persistence of this short‐lived toad in ephemeral systems are of great concern. This in addition to invasive species, habitat alteration, and hydrological pressures on remaining populations in perennial systems threatens the long‐term persistence of this species.

Many aquatic and semi‐aquatic species adapted to southwestern and Mediterranean ecosystems are similarly at risk from reduced surface water availability, leading to reduced aquatic connectivity, recruitment, and survivorship (Jaeger et al., 2014; Jones et al., 2017; Leidy, Bogan, Neuhaus, Rosetti, & Carlson, 2016; Lovich et al., 2017; Montgomery et al., 2015). For species that burrow and forage in adjacent terrestrial systems, increased drought stress can further affect overwinter survival and prey availability due to reduced soil moisture and associated plant and animal mortality (Lovich et al., 2017; Venturas et al., 2016). The arroyo toad serves as a model for understanding semi‐aquatic species' responses to long‐term climatic and hydrologic changes and the potential loss of biological integrity from these unique Mediterranean stream systems. Freshwater‐dependent species could benefit from adaptive management strategies that rebuild resiliency in the face of projected changes in climate and water availability.

## CONFLICT OF INTEREST

None declared.

## AUTHOR CONTRIBUTIONS

RNF, SAH, TEH designed study and helped with implementation. MLW, DCS led field data collection. SAH managed data and performed preliminary analyses. CSB performed formal statistical analyses and produced figures. CSB and RNF did the writing and interpretation. All others participated in interpretation, review and edits to the manuscript.
